# The FMN “140s Loop” of Cytochrome P450 Reductase Controls Electron Transfer to Cytochrome P450

**DOI:** 10.3390/ijms221910625

**Published:** 2021-09-30

**Authors:** Freeborn Rwere, Sangchoul Im, Lucy Waskell

**Affiliations:** 1 Department of Anesthesiology, University of Michigan and VAMC, 2215 Fuller Road, Ann Arbor, MI 48105, USA; scim@med.umich.edu (S.I.); waskell@umich.edu (L.W.); 2 Department of Anesthesiology, Perioperative and Pain Medicine, Stanford University School of Medicine, 300 Pasteur Drive, Stanford, CA 94305, USA; 3 Department of Internal Medicine, University of Michigan and VAMC, 2215 Fuller Road, Ann Arbor, MI 48105, USA

**Keywords:** cytochrome P450 reductase, cytochrome P450, cytochrome *b5*, redox potential, anionic semiquinone, hydroquinone, P450 BM3

## Abstract

Cytochrome P450 reductase (CYPOR) provides electrons to all human microsomal cytochrome P450s (cyt P450s). The length and sequence of the “140s” FMN binding loop of CYPOR has been shown to be a key determinant of its redox potential and activity with cyt P450s. Shortening the “140s loop” by deleting glycine-141(ΔGly141) and by engineering a second mutant that mimics flavo-cytochrome P450 BM3 (ΔGly141/Glu142Asn) resulted in mutants that formed an unstable anionic semiquinone. In an attempt to understand the molecular basis of the inability of these mutants to support activity with cyt P450, we expressed, purified, and determined their ability to reduce ferric P450. Our results showed that the ΔGly141 mutant with a very mobile loop only reduced ~7% of cyt P450 with a rate similar to that of the wild type. On the other hand, the more stable loop in the ΔGly141/Glu142Asn mutant allowed for ~55% of the cyt P450 to be reduced ~60% faster than the wild type. Our results reveal that the poor activity of the ΔGly141 mutant is primarily accounted for by its markedly diminished ability to reduce ferric cyt P450. In contrast, the poor activity of the ΔGly141/Glu142Asn mutant is presumably a consequence of the altered structure and mobility of the “140s loop”.

## 1. Introduction

Cytochrome P450 reductase is a ~78 kDa diflavin oxidoreductase protein that delivers electrons to microsomal cytochromes P450, heme oxygenase, and cytochrome *b5* [1,2,3]. Flavo-protein catalyzed reactions are versatile because of the presence of a unique flavin isoalloxazine ring system that can undergo redox linked chemical reactions [4,5]. Microsomal cytochromes P450, functioning as monooxygenases, catalyze the oxidative biotransformation of the majority of pharmaceuticals in current use, carcinogens and pro-carcinogens, fatty acids, and steroids [6,7,8]. CYPOR can provide the two electrons needed for the oxidation of different substrates, whereas cyt *b5* can only provide the second required electron [1,2]. The ability of CYPOR to donate electrons to P450s depends on the redox potential of its flavin cofactors, FMN and FAD. The redox potential of wild-type CYPOR for the FMN_ox/sq_ couple is found at −56 mV, whereas the FMN_sq/hq_ couple appears at −252 mV [9]. It is the low potential FMN hydroquinone that reduces microsomal cyts P450 heme. The structure and dynamics of the “140s” FMN loop of CYPOR (140-YGEGD-144) play a critical role in electron transfer to microsomal cyt P450s. In contrast, in the model bacterial cyt P450, BM3, where the heme-binding domain and flavin domain are fused in a single polypeptide chain, the redox potential of the oxidized/semiquinone FMN_ox/sq_ couple appears at −240 mV, whereas the semiquinone/hydroquinone FMN_sq/hq_ couple is found at −160 mV [10]. It is the more-negative, low potential, unprotonated, anionic semiquinone (−240 mV) that can reduce the P450 BM3 heme. The function of the “537s” loop (537-YNGH-540) of P450 BM3 is analogous to that of the “140s” loop of CYPOR. In this work, the CYPOR “140s” loop has been engineered (shortened by one amino acid and changed amino acid sequence) to resemble that of the “537s” FMN binding loop of P450 BM3 [9]. The engineered ΔGly-141 mutants have redox properties similar to the flavo-cytochrome P450 BM3 but different from wild-type CYPOR. For example, the redox potentials of both ΔG141 and ΔG141/E142N mutants were reversed from those of wild-type CYPOR but similar to P450 BM3, that is the ox/sq mutant couple was lower, more negative, than the sq/hq couple. The ΔG141 redox potentials appeared at −229 mV for the FMN_ox/sq_ couple and −53 mV for the FMN_sq/hq_ couple, whereas those for ΔG141/E142N were found at −281 mV (FMN_ox/sq_) and −204 mV (FMN_sq/hq_) [9].

The redox potential of substrate-bound, ferric cyt P450 2B4 is approximately −245 mV [11]. Based on its redox potential (−281 mV) for FMN_ox/sq_, the anionic semiquinone of the ΔG141/E142N is thermodynamically competent to transfer electrons to substrate-bound ferric cyt P450 2B4. The ΔG141 mutant with a redox potential of −229 mV for FMN_ox/sq_ should at least partially reduce ferric P450. Cyt *b5* (~25 mV) cannot reduce ferric cytochrome P450 but can transfer the second electron to the high potential oxy-ferrous cytochrome P450 [12]. To better understand the molecular basis of the previously reported mutants’ low activity with cyt P450, we asked whether the mutants could reduce ferric cyt P450 and whether cyt *b5* would be able to restore the activity of ΔG141 and ΔG141/E142N mutants by reducing oxyferrous cyt P450. In other words, can activity be increased by having the anionic semiquinone of ΔG141 or ΔG141/E142N mutant transfer the first electron to cyt P450 2B4, and then can cyt *b5* provide the second electron and restore the activity of the mutant reductases?

## 2. Results

### 2.1. Comparison of the Kinetics of Reduction of Ferric Cytochrome P450 by Wild Type and Mutant Reductases

**Figure 1:**
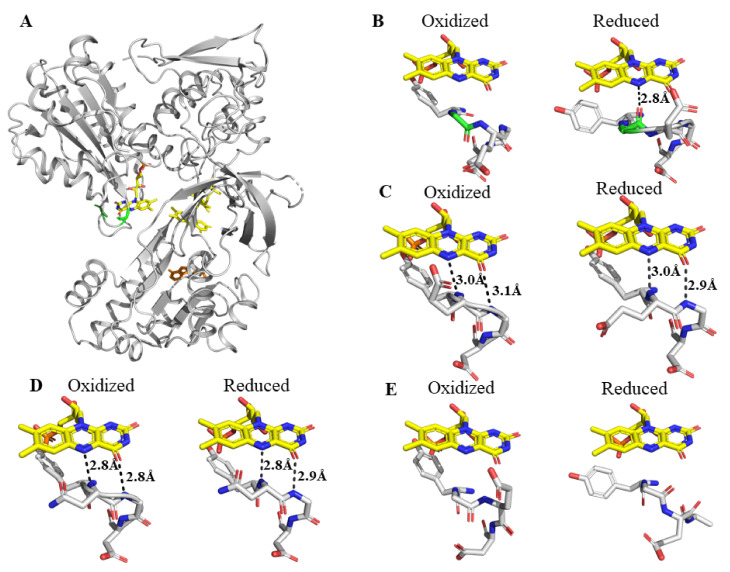
Comparison of the structure of the “140s loop” in the wild-type CYPOR and ΔG141, ΔG141/E142N and ΔG143 mutants. The 140s loop controls the FMN redox potential and electron transfer to P450s. (**A**) Structure of the oxidized wild-type CYPOR (PDB ID: 4YAF), with two amino acids within the structure highlighted, Gly141 (light green) and Gly143 (dark green). (**B**) Wild-type oxidized (ox) (PDB ID: 4YAF) and reduced (red) (PDB ID: 4YAL) 140s loop. Upon reduction, the loop flips up toward the flavin and forms a stable hydrogen bond (H-bond) between the protonated flavin nitrogen 5 (N5H) and the Gly141 carbonyl oxygen. (**C**) The shorter ΔG141 oxidized (PDB ID: 4Y9R) and reduced (PDB ID: 4YAW) loop, which is closer to the flavin than in the wild-type protein, sterically hinders N5 protonation and forms 2 H-bonds between amide nitrogens of Glu142 and Gly143 and the N5 and O4 of the FMN ring, respectively. (**D**) The shorter ΔG141/E142N oxidized (PDB ID: 4Y7C) and reduced (PDB ID: 4YAU) loops form two relatively strong H-bonds between the amide nitrogens of Asn142 and Gly143 with N5 and O4 of the FMN ring, respectively. (**E**) The ΔG143 oxidized (PDB ID: 4Y9U) and reduced (PDB ID: 4YAO) loop is highly disordered and mobile with a higher average B-factor than the average B-factor of the whole CYPOR.

The location of Gly141 and G143 residues in the “140s loop” of oxidized wild-type rat cytochrome P450 reductase is shown in Figure 1A. In its oxidized form, the “140s loop” of CYPOR forms a Gly141–Glu142 peptide bond adopting an O-down conformation, which rotates to an up position in the reduced protein (Figure 1B). In the wild type reduced CYPOR, the backbone carbonyl oxygen of Gly141 hydrogen bonds to the hydrogen on nitrogen 5 (N5) in the reduced flavin, thereby stabilizing the uncharged-neutral, blue semiquinone (Figure 1B). Upon Gly141 deletion (ΔG141), the amide nitrogen of Glu 142 in the shortened “140s loop” is so close to the FMN N5 atom that it sterically hinders the flavin N5 protonation. As a result of the unfavorable protonation of N5, the one-electron reduced flavin forms a negatively-charged anionic semiquinone that leads to a decrease in its potential. Furthermore, the reduced form of ΔG141 cannot (unlike the reduced wild type) undergo a peptide bond flip that creates the stabilizing interaction between the flavin N5 and backbone C=O, carbonyl. The main chain amide nitrogens of Glu142 and Gly143 make two H-bonds with nitrogen 5, N5, and oxygen, O4, of the FMN ring, respectively (Figure 1C). When Gly141 is deleted and Glu142 mutated to Asn142 (ΔG141/E142N) in order to mimic the sequence of the P450 BM3 FMN loop, the backbone amide nitrogen of Asn142 and Gly143 form H-bonds with the unprotonated N5 and O4 of the FMN ring. These H-bonds decrease the mobility of the mutants’ loop (Figure 1D). Recall that, in the wild type, the flavin N5 becomes protonated upon reduction and the G141 backbone oxygen flips up toward the flavin and H-bonds with hydrogen on N5 while the amide nitrogen of G143 still H-bonds with the flavin O4. The shortening of the loop in the G141 deletion mutants, which brings the residues in the loop closer to the flavin, allows the amide of the amino acid (glutamic acid or asparagine) following the missing glycine to H-bond with the unprotonated N5 in the oxidized flavin and sterically prevent protonation upon reduction. In the shortened, very mobile loop of the ΔG143 structure, no H-bonds between the flavin and the loop are observed. However, the properties and activity of the ΔG143 indicate that H-bonds similar to those in the wild type are formed transiently (Figure 1E).

**Figure 2:**
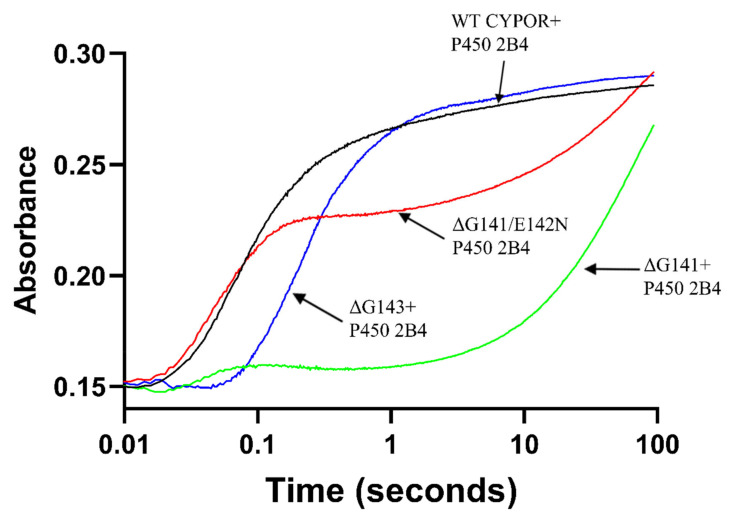
Kinetics of the reduction of ferric cyt P450 2B4 by WT CPR, ΔG141, ΔG141/E142N and ΔG143 in the presence of 10-fold excess of NADPH monitored at 450 nm. The experiment was conducted under anaerobic conditions at pH 7.4 and 30 °C.

In an attempt to determine whether the anionic semiquinone of the ΔGly-141 reductase mutants can reduce ferric cyt P450 2B4, we investigated the rate of first electron transfer to ferric cyt P450. By monitoring the absorbance increase at 450 nm as a result of formation of a ferrous cyt P450-CO adduct, we can determine whether the mutants deliver electrons to P450s [13]. Figure 2 and Table 1 show the rate and extent of reduction of ferric cyt P450 2B4 by wild-type CYPOR and the glycine deletion mutants under conditions similar to those employed to measure activity. The reduction of cyt P450 2B4 (−245 mV) by the FMN hydroquinone (−252 mV) of wild-type CYPOR when monitored at 450 nm is biphasic with the biphasic rate constants k_1_ = 9.93 s^−1^ and k_2_ = 0.54 s^−1^. The fast phase (k_1_ = 9.93 s^−1^) represents the transfer of electrons from the FMN hydroquinone to approximately 80% of the ferric cyt P450 [13]. In the fast phase of the biphasic reduction of ferric cyt P450 2B4 by the ΔG143 mutant (k_1_ = 3.34 s^−1^ and k_2_ = 0.11 s^−1^), approximately 60% of the ferric P450 is reduced approximately 66% slower than those of wild-type CYPOR (Table 1 and Table 2). In contrast, the reduction of ferric cyt P450 2B4 by the ΔG141/E142N mutant produced two distinct phases, a fast phase (k_1_ = 16.29 s^−1^) and a very slow phase (k_2_ = 0.03 s^−1^). The fast phase (k_1_ = 16.29 s^−1^) represents electron transfer from the moderately stable, low-potential anionic FMN semiquinone to ferric cyt P450. Since the anionic semiquinone is short lived at pH 7.4 (t_1/2_ < 0.1 s), it was only able to reduce ~55% of the ferric cyt P450 2B4 [9]. The source of the electrons for the slower phase of reduction is not understood at this time. The reduction of ferric cytP450 2B4 by the ΔG141 mutant was fitted to a triphasic exponential function with a small fast phase (k_1_= 9.12 s^−1^) and two larger amplitude slow phases (k_1_ = 0.03 s^−1^ and k_2_ = 0.01 s^−1^) (Table 1, Figure 2 and Figure S1). The fast phase (k_1_ = 9.12 s^−1^) with a small amplitude (~7%) presumably represents electron transfer from the transient anionic semiquinone to 7% of ferric cyt P450 2B4.

### 2.2. Effect of Cytochrome b5 on the Rate of Benzphetamine Metabolism by Cyt P450 2B4 under Steady-State Conditions at 30 °C in the Presence of Wild-Type and Mutant Reductases

In an effort to understand the biochemical basis of the inability of the glycine deletion mutants to support cyt P450 catalysis, we asked whether cyt *b5* could increase the activity of the Gly141 mutants by providing the second electron to oxyferrous P450 after the mutants provided the first electron to ferric cyt P450. As shown in Table 2, the activity of wild-type CYPOR was modestly enhanced at a low concentration of cyt *b5* (0.68 vs. 0.82 nmol/s/nmol of CYPOR). The maximum increase in activity of wild-type CYPOR was achieved at molar ratios of 1:0.5 for CYPOR and cyt *b5*, respectively. At high concentrations of cyt *b5*, the activity of P450 2B4 was greatly reduced because cyt *b5* occupies the CYPOR binding site on the proximal surface of P450 2B4 and prevents a reduction of ferric P450 (Table 2) [12]. Since cyt *b5* can enhance the activity of reductases at low concentrations, the glycine deletion mutants were also examined for their ability to support metabolism of benzphetamine in the presence of various concentrations of cyt *b5* in order to investigate whether cyt *b5* can augment the activity of these mutant reductases. As shown in Table 2, the ΔG141 and ΔG141/E142N mutants are essentially inactive in the absence of cyt *b5*. Addition of cyt *b5* at various concentrations did not significantly enhance the activity. The activity of ΔGly143 mutant was slightly enhanced at a lower molar ratio of cyt *b5* (0.08 vs. 0.11 nmol/s/nmol of ΔG143 CYPOR). However, at higher cyt *b5* concentrations, the activity of ΔG143 is reduced significantly.

## 3. Discussion

Structural differences between CYPOR/P450 2B4 and flavo-cytochrome P450 BM3 from the *Bacillus megaterium* system account for their differences in redox potentials and reactivity. In this work, we deleted a conserved Gly141 and mutated Glu142 to asparagine in order to mimic the P450 BM3 FMN binding loop. We also deleted glycine 143 in order to test whether a shortened and less flexible “140s” loop can reduce ferric P450 2B4. Our results showed that ΔG141 can transfer the first electron to ferric CYP2B4 at a rate approximately equal to the wild type, whereas ΔG141/E142N reduces ferric CYP2B4 at a rate ~1.6-fold higher than the wild type, consistent with its more negative potential, thermodynamically favorable anionic semiquinone. The amount of heme reduced by anionic semiquinone was significantly different between ΔGly141 and ΔG141/E142N (~7% for ΔG141 vs. 55% for ΔG141/E142N). Both ΔGly141 and ΔG141/E142N mutants were inactive with CYP2B4, and their activities were not significantly stimulated by cytochrome *b5*. The increase in rate of heme reduction and greater amount of heme reduced by ΔG141/E142N vs. ΔG141 can be attributed to a more rigid “140s” FMN loop and a greater thermodynamic driving force, i.e., a lower potential.

A closer look at the crystal structures of the reduced ΔGly-141 mutants reveals that ΔG141 and ΔG141/E142N form two H-bonds between E142 or N142 and G143 with N5 and O4 of the FMN (Figure 1C,D). The mobility of their “140s” loop is considered different. The crystal structure suggests that, in some conformations the 5 carbon long glutamic acid can sterically collide with the flavin ring, which prevents stable intramolecular interactions. In contrast, a shorter four-carbon asparagine side chain at position 142 does not collide with the flavin and consequently forms more stable interactions in the molecule. The more stable loop and low potential anionic semiquinone in the ΔG141/E142N structure versus a dynamic loop and higher potential anionic semiquinone in the ΔG141 structure allows ferric P450 to be significantly and quickly reduced versus ΔG141 where the higher potential anionic semiquinone quickly decays to the hydroquinone with a potential of ~−53 mV, which cannot reduce ferric cyt P450. It should also be noted that the hydroquinone is more negative in the E142N protein again a reflection of a stable loop. Another difference between the ΔG141/E142N and the analogous FMN domain loop of flavo-cytochrome P450 BM3 is that the P450 BM3 loop ends with two rigid proline residues that essentially immobilize the loop. In this conformation, the amide proton of Asn-537 in P450 BM3 forms a strong H-bond with N5 atom of the FMN isoalloxazine ring, while Thr577 forms another H-bond with a keto O4 of FMN. This more stable and less dynamic FMN P450 BM3 loop allows the anionic semiquinone to reduce the heme at significantly higher rates compared with the engineered ΔGly-141 mutants. Flavo-cytochrome P450 BM3 oxidizes substrates at high turnover rates of >1000/min [14]. A comparison of the structure of the flavo-cytochrome P450 BM3 system with the CYPOR/cyt P450 2B4 system reveals that the lack of enzymatic activity of the ΔG141/E142N mutant is due to the decreased stability of anionic semiquinone and the relatively longer time required for the mutant CYPOR to bind to cyt P450. In the two-separate-protein CYPOR/cyt P450 2B4 system, the two proteins must come into contact prior to heme reduction; in contrast, in the P450 BM3 system, the reductase and cyt P450 are fused in a single polypeptide chain.

The ΔG143 mutant reduced substrate-bound ferric cyt P450 2B4 at a significantly slower rate compared with wild-type CYPOR. The amount of heme reduced by this mutant was comparable with wild-type CYPOR. However, as shown in Table 2, the activity of ΔG143 is significantly lower than that of the wild type and is slightly enhanced by cytochrome *b5*. The markedly reduced activity of the ΔG143 mutant with cyt P450 2B4 under steady-state conditions (Table 2) is likely due to slower rate of reduction and the presence of a flexible FMN “140s loop”, which prevents tight docking with cyt P450 2B4. The interaction between the mutant reductases, and ferric and oxyferrous P450 may be different, with the oxyferrous protein having more stringent structural requirements for reduction and catalysis. It has been reported that P450 camphor has more specific requirements for reduction of the oxyferrous compared to the ferric protein, and this may also be the case with P4502B4 [15]. The biochemical basis for the slight stimulation of the ΔG143 mutant at lower concentrations of cyt *b5* is likely due to its ability to increase the rate of proton delivery to the hydroperoxo (Fe^3+^OOH)^−^ intermediate and thereby to enhance formation of the oxyferryl (Fe^4+^O) π cation radical, Compound I, and to decrease side product (H_2_O_2_) formation [16]. The inhibitory effects of cytochrome *b5* on activity of ΔG143 at higher concentrations are presumably due to competition between the reductases and cyt *b5* for the binding site on the proximal surface of P450 2B4 [12].

In summary, the results presented here demonstrate for the first time that an anionic semiquinone of the ΔG141/E142N mutant can transfer the first electron to substrate-bound ferric cyt P450 2B4 at an initial rate ~1.6 times faster than that of the FMN hydroquinone of wild-type CYPOR. This may be due to a ~50 mV greater thermodynamic driving force (ΔG141/E142 −281 mV ox/sq vs. wild type −252 mV sq/hq). The more transient anionic semiquinone of ΔG141 mutant also reduced ~7% of ferric cytochrome P450 2B4, slightly slower than the wild-type reductase. Our results also indicate that the length and dynamics of the FMN loop of CYPOR are major determinants of electron transfer to microsomal P450s. Finally, the activity of the ΔGly-141 mutants with cyt P450 was not enhanced by the presence of cytochrome *b5*.

## 4. Materials and Methods

### 4.1. Construction of Site-Directed Mutants of the Full-Length CYPOR

Construction of site-directed mutants of the full-length rat CYPOR (ΔG141, ΔG141/E142N and ΔG143) were according to guidelines from the QuikChange II XL site directed mutagenesis kit (Agilent Technologies) as previously described [9]. The oligomers for site-directed mutagenesis are presented in Table 3. The mutated DNA was sequenced at the University of Michigan DNA Sequencing Core Facility.

### 4.2. Expression and Purification of the Full-Length Wild-Type (WT) and Glycine Deletion CYPOR Mutants

The expression of full-length wild-type and mutant CYPOR was conducted according to the published procedures [9,17] in the presence of 0.24 mM carbenicillin, 0.2% (w/v) glucose and 5.3 nM riboflavin. For induction, 0.4 mM IPTG (isopropylthio-β-galactoside) was used. The full-length wild-type CYPOR and glycine deletion mutants were purified as previously described [9,17]. A 10% SDS gel was run on purified proteins, and they all gave a single band at ~74 kDa. The average yield of pure full-length wild-type and mutant reductase proteins was ~20–25 mg/L.

### 4.3. Measurement of Benzphetamine Metabolism by Cyt P450 2B4

Wild-type rabbit cyt P450 2B4 and rabbit cyt *b5* were expressed and purified as previously described [12,13,18]. The metabolism of benzphetamine was carried out at 30°C under steady-state conditions by mixing various concentrations of microsomal cyt *b5* (0, 0.1, 0.2, 0.3 and 0.4 μM) and a fixed concentration of cyt P450 2B4 (0.2 µM) and CYPOR (0.2 µM) in the presence of DLPC (225 µM), and incubating the mixture for 1 h at room temperature. The solution was then diluted to 0.8 mL with 50 mM potassium phosphate buffer, pH 7.4, containing 1 mM benzphetamine. The resultant mixture was equilibrated for 5 min at 30 °C, and the reaction was initiated by adding NADPH (300 µM final concentration). The reaction was allowed to proceed for 6 min at 30 °C. After 6 min, the reaction was quenched immediately with 70% trichloroacetic acid. The quenched reaction mixtures were centrifuged at 13,000 x g for 5 min, and 200 mL of supernatant was assayed for formaldehyde using Nash’s reagent according to the published protocols and the amount of formaldehyde formed from metabolism of benzphetamine by cyt P450 2B4 was calculated from the calibration curve. [13,18,19,20]. The values in Table 2 are an average of three measurements on two separate days.

### 4.4. Kinetics of the Reduction of Ferric Cyt P450 2B4 by CYPOR

The rate of electron transfer from wild-type or mutant CYPOR to ferric cyt P450 2B4 was measured at 30 °C with stopped-flow spectrophotometry by monitoring the absorbance increase at 450 nm as a result of formation of the ferrous cyt P450-CO adduct. The stopped-flow experiments were performed using a Hi-Tech SF61DX2 stopped-flow spectrophotometer (Hi-Tech, Wiltshire, UK) housed in an anaerobic chamber (Belle Technology, Dorset, UK) as reported previously [13]. A solution containing 5 µM P450 2B4 and 5 μM CYPOR in 0.1 M potassium phosphate, pH 7.4, 15% glycerol, 300 μM DLPC, and 1 mM benzphetamine was prepared under anaerobic conditions. The anaerobic protein mixture was rapidly mixed with a carbon monoxide saturated 0.1 M potassium phosphate buffer, pH 7.4, containing 15% glycerol, 1 mM benzphetamine, and 50 μM NADPH at 25 °C. The absorbance change at 450 nm was recorded as a function of time. The rate constants and amplitudes were obtained by fitting the absorbance change at 450 nm with a double exponential function (nonlinear curve fit) for wild-type, ΔG141/E142N and ΔG143 using KinetAsyst2 software (Hi-Tech) and GraphPad Prism. A triple exponential function was used for ΔG141 mutant, and the kinetic parameters are shown in Table 1.

## Figures and Tables

**Table 1:** Kinetics of the reduction of substrate-bound ferric cyt P450 2B4 by WT CYPOR and G141 loop mutants in the presence of a 10-fold excess of NADPH at pH 7.4.

	λ= 450 nm			
Enzyme	k_1obs_ (s^−1^) (amplitude%)	k_2obs_ (s^−1^) (amplitude%)	k_3obs_ (s^−1^) (amplitude%)	R^2^
WT CPR	9.93 ± 0.17 (82.8 ± 0.2)	0.54 ± 0.014 (17.2 ± 0.2)		0.9951
ΔG141	9.12 ± 0.10 (7.2 ± 0.3)	0.03 ± 0.001 (89.2 ± 0.6)	0.01 ± 0.001 (3.6 ± 0.2)	0.9994
ΔG141/E142N	16.29 ± 0.31 (60.1 ± 0.8)	0.03 ± 0.001 (39.9 ± 0.8)		0.9938
ΔG143	3.34 ± 0.10 (81.8 ± 0.1)	0.11 ± 0.01 (18.2 ± 0.1)		0.9943

**Table 2:** Effect of cyt *b5* on the rate of benzphetamine metabolism by cyt P450 2B4 under steady-state conditions, as described in the “Materials and Methods” section.

Full-Length CYPOR Reductase Mutant/Wild-Type	Molar Ratio P450:CYPOR:Cyt *b5*	Benzphetamine Nmol of CH_2_O Produced/s/nmol CYPOR
		
WT	1:1:0	0.68 ± 0.01
	1:1:0.5	0.82 ± 0.04
	1:1:1	0.73 ± 0.04
	1:1:1.5	0.63 ± 0.01
	1:1:2	0.52 ± 0.01
	1:1:5	0.26 ± 0.02
		
ΔG141	1:1:0	0.004 ± 0.001
	1:1:0.5	0.01 ± 0.003
	1:1:1	No activity
	1:1:1.5	No activity
	1:1:2	No activity
		
ΔG141/E142N	1:1:0	0.001 ± 0.0006
	1:1:0.5	0.01 ± 0.003
	1:1:1	No activity
	1:1:1.5	No activity
	1:1:2	No activity
		
ΔG143	1:1:0	0.08 ± 0.005
	1:1:0.5	0.11 ± 0.003
	1:1:1	0.07 ± 0.011
	1:1:1.5	0.05 ± 0.002
	1:1:2	0.03 ± 0.001

**Table 3:** Sequences of oligonucleotide primers used to mutate full length rat cyt P450 reductase (CYPOR).

Mutant	Sequence
ΔG141 (Forward)	5′-TGC ATG GCC ACA TAC --- GAG GGC GAC C-3′
ΔG141/E142N (Forward)	5′-TGC ATG GCC ACA TAC --- AAC GGC GAC C-3′
ΔG143 (Forward)	5′-ATG GCC ACA TAC GGA GAG --- GAC CCC A-3′

## Data Availability

Not applicable.

## Supplementary Materials

Supplementary materials can be found at www.mdpi.com/article/10.3390/ijms221910625/s1.

## Abbreviations

Cytochrome P450 reductase, CYPOR; wild type, WT; cytochrome *b5*, cyt *b5*; cytochrome P450, P450 or cyt P450; flavo-cytochrome P450 BM3, P450 BM3; oxidized, ox; reduced, red; semiquinone, sq; hydroquinone, hq.
