# Supplementary material for: The FMN “140s Loop” of Cytochrome P450 Reductase Controls Electron Transfer to Cytochrome P450
Source: Int J Mol Sci. 2021 Sep 30;22(19):10625. doi: 10.3390/ijms221910625 (PMC8508823; doi:10.3390/ijms221910625)
Supplement: Supplementary file 1 [file ijms-22-10625-s001.zip › ijms-1383731-supplementary.pdf]

Communication

The FMN "140s loop" of cytochrome P450 reductase controls electron transfer to Cytochrome P450

Freeborn Rwere[1,2*], Sangchoul Im[1,3] and Lucy Waskell[1]

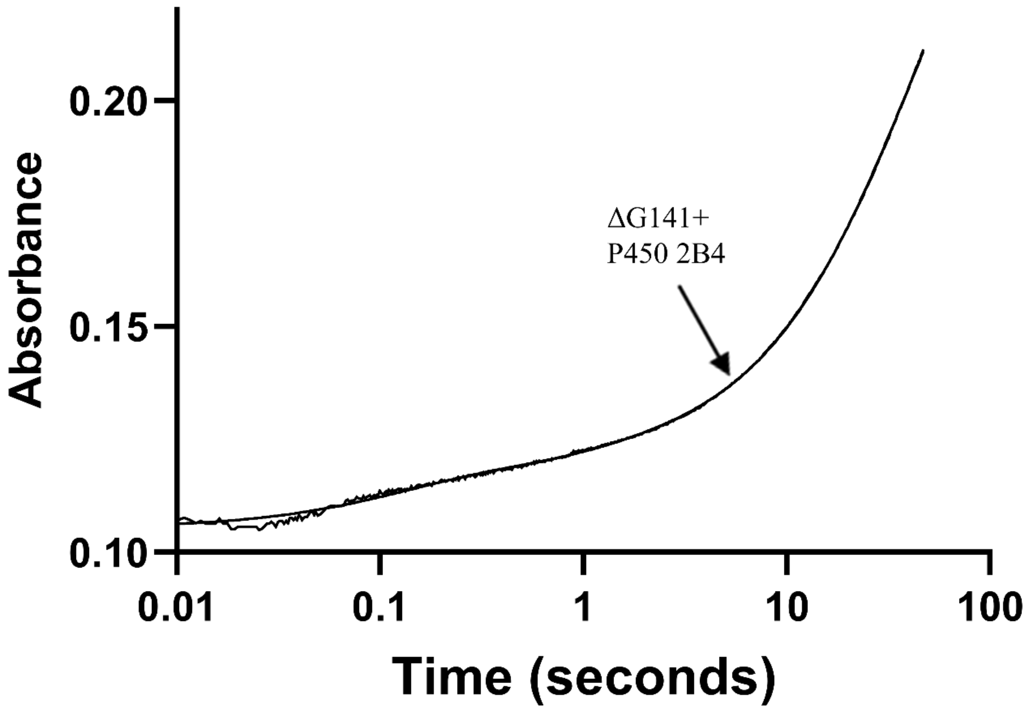

Supplementary

Figure S1. Kinetics of the reduction of ferric cyt P450 2B4 by ΔG141 CYPOR in the presence of 10-fold excess NADPH monitored at 450 nm. The experiment was conducted under anaerobic conditions at pH 7.4 and 30°C. The data were fitted with a triphasic function.

Supplementary Curve Fitting Data for the ΔG141 mutant

Three-phase decay	
Best-fit values	
Y0	0.1053
Plateau	0.2503
Percent Fast	7.192
K_{Fast}	8.764
Percent Slow	89.30
K_{Slow}	0.02517
K_{medium}	1.052
Half Life (Slow)	27.54
Half Life (Fast)	0.07909
Half Life (Medium)	0.6590
95% CI (profile likelihood)	
Y0	0.1051 to 0.1056
Plateau	0.2484 to 0.2525
Percent Fast	6.732 to 7.606
KFast	7.943 to 9.699
Percent Slow	89.10 to 89.50
KSlow	0.02445 to 0.02584
Kmedium	0.8100 to 1.321
Half Life (Slow)	26.82 to 28.35
Half Life (Fast)	0.07146 to 0.08727
Half Life (Medium)	0.5246 to 0.8557
Goodness of Fit	
Degrees of Freedom	630
R squared	0.9994
Sum of Squares	0.0002525
